# Mechanotransduction mechanisms in human erythrocytes: Fundamental physiology and clinical significance

**DOI:** 10.1080/19336950.2025.2556105

**Published:** 2025-09-10

**Authors:** Lennart Kuck, Lars Kaestner, Stéphane Egée, Virgilio L. Lew, Michael J. Simmonds

**Affiliations:** a Molecular Cardiology and Biophysics Division, Victor Chang Cardiac Research Institute, Sydney, New South Wales, Australia; b Theoretical Medicine and Biosciences, Medical Faculty, Saarland University, Homburg, Germany; c Dynamics of Fluids, Experimental Physics, Faculty of Natural Science and Technology, Saarland University, Saarbrücken, Germany; d Biological Station Roscoff, Sorbonne University, CNRS, UMR8227, Laboratory of Integrative Biology and Marine Models, Roscoff, France; e Laboratory of Excellence Globule rouge, Biogenèse et Pathologies du Globule Rouge, Paris, France; f Physiological Laboratory, Department of Physiology, Development and Neuroscience, University of Cambridge, Cambridge, UK; g Biorheology Research Laboratory, Faculty of Health, Griffith University, Gold Coast, Australia

**Keywords:** Mechanotransduction, Piezo1, red blood cell, biophysics, blood, membrane

## Abstract

The hallmarks of mechanosensitive ion channels have been observed for half a century in various cell lines, although their mechanisms and molecular identities remained unknown until recently. Identification of the bona fide mammalian mechanosensory Piezo channels resulted in an explosion of research exploring the translation of mechanical cues into biochemical signals and dynamic cell morphology responses. One of the Piezo isoforms – Piezo1 – is integral in the erythrocyte (red blood cell; RBC) membrane. The exceptional flexibility of RBCs and the absence of intracellular organelles provides a unique mechanical and biochemical environment dictating specific Piezo1-functionality. The Piezo1-endowed capacity of RBCs to sense the mechanical forces acting upon them during their continuous traversal of the circulatory system has solidified a brewing step-change in our fundamental understanding of RBC biology in health and disease; that is, RBCs are not biologically inert but rather capable of complex dynamic cellular signaling. Although several lines of investigation have unearthed various regulatory mechanisms of signaling pathway activation by RBC-Piezo1, these independent studies have not yet been synthesized into a cohesive picture. The aim of the present review is to thus summarize the progress in elucidating how Piezo1 functions in the unique cellular environment of RBCs, challenge classical views of this enucleated cell, and provoke developments for future work.

## Erythrocyte physiology

Circulating erythrocytes (red blood cells; RBC) account for over 80% of all human cells and are continuously exposed to dynamic mechanical stresses over their lifespan. Despite this, much of what is commonly known about RBC originates from the field of hematology which relies on observing cell counts, morphology, and increasingly proteomic profiles evaluated in static models that negate *in vivo* dynamics. Indeed, so vital is mechanical stimulation to these cells that differentiation and maturation of precursors (e.g. transition from erythroblasts to reticulocytes) appear intimately influenced by shear forces [1]. The importance of exploring RBC in such dynamic environments is not trivial: hematopoietic tissues are responsible for their continuous production at a daily rate of ~ 1% total cell count – it is thus reasonable to estimate that in adult males ~ 3 million RBC are turned over every second. Curiously, maturation of precursors into circulating RBC which occurs so effortlessly *in vivo* has been remarkably challenging to replicate *in vitro*: to date, cultured RBC have been profoundly limited in quantity and quality [2]. Accumulating evidence demonstrates that enhanced kinetics of differentiation may be achieved using even simple (and poorly controlled) orbital shakers [1], while phosphorylation events that occur when reticulocytes are deformed appear distinct of those in mature RBC, and represent a functionally important element of maturation [3]. During these maturation stages, erythroblasts physically extrude their nuclei and internal organelles, while degradation of internal organelles, polyribosomes, and several structural proteins by reticulocytes signals the ultimate transition toward mature RBC. These maturation steps induce some limitations but also functional benefits: while mature RBC lack the machinery to synthesize proteins and thus are susceptible to environmental stressors, the resultant biophysical properties promote rheological advantages. Specifically, the final transition from reticulocyte to mature RBC reduces intracellular viscosity, partly driven by lipid remodeling [4], and produces a final morphology that has approximately 50% greater surface area than a sphere of equivalent volume, factors which facilitate an exceptional membrane flexibility among all mammalian cells. This deformability has a profound impact on microcirculatory flux, given the ~8 μm cell must pass capillaries of only 2–3 μm diameter [5].

**Figure 1.**
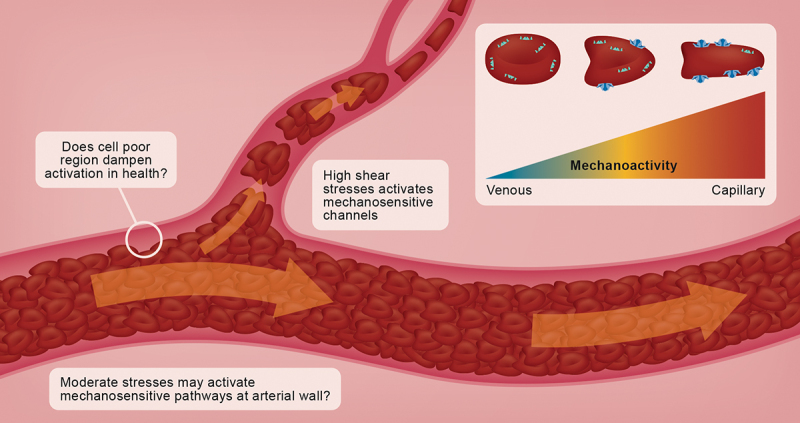
The physical properties of red blood cells (RBC) influence their position in flow and govern interactions with the vascular wall through facilitating a cell-poor region. The cell-poor region, and the associated moderate shear stresses within larger vessels, may dampen membrane strain and thus Piezo1 activation in health, but it is clear that the higher shear regions of resistance vessels are sufficient to alter the morphology of RBC, and the corresponding membrane strain activates a proportion of the Piezo1 pool (active Piezo1 represented by dark blue and “larger” channels for illustrative purposes only).

The unique physical properties of RBC facilitate several important processes that optimize their primary functions. At a bulk fluid level, flexible particles such as RBC may align with the direction of flow and adopt optimal hemodynamic morphologies (e.g. croissant and slipper shaped) [6,7], which ultimately reduces the fluid’s internal resistance to flow (i.e. viscosity). This means that rather than blood exhibiting a singular viscosity (per Newtonian fluids like water), blood viscosity decreases when it is exposed to high shear rates typical of the arterial network, in part, due to deformation of the highly flexible RBC. Further, the dynamic morphological shifts that occur in flowing RBC results in an accumulation of these cells in the central axis of flow, and thus also a cell-poor region at the vessel wall [8]. This axial migration of RBC lends to reduced wall shear stress, marginates platelets and leukocytes to the vessel wall, and also promotes enhanced perfusion for a given driving pressure. As blood approaches the evermore narrow geometries of the microcirculation, some peculiar observations are made: rather than flow slowing due to the expected increase in resistance, a decrease in apparent viscosity results in increased blood flow. In addition to the axial migration outlined above, the phase separation of plasma from cells results in the cell-poor layer serving as a lubricating sleeve, particularly at geometries approaching unity with the cell diameter. Figure 1 provides a simplified model of the rheological, cellular and biophysical properties that govern these processes. The fact that these observations may be abolished through rigidification of the RBC membrane highlights the importance of cell deformation to perfusion of the microcirculation [9].

Classically, the processes outlined above were thought to occur in a purely unidirectional manner; that is, the RBC was modeled as little more than a hemoglobin carrier with physical attributes, such as morphology and deformation, that were passively determined by its environment. Perhaps the lack of molecular machinery in RBC led to this simplistic view, although this model has been fundamentally undermined over the past decades owing to an accumulation of evidence that these cells sense their environment, potentially regulate their own physical properties, modulate vascular tone, and ultimately govern local blood flow and gas transport [for review, see 8]. Profound early evidence contributing to the contemporary view includes several physical signals, including decreased pH, increased PCO_2_, and increased temperature, but also mechanical stress [10], stimulate the release of ATP from RBC. ATP release from RBC requires several interacting signaling processes that, once activated, ultimately leads to the vasodilation of adjacent vasculature and thus also regulation of tissue perfusion [11]. Subsequent works led to the finding that, in addition to well-described nitrite sources of nitric oxide (NO), mature RBC contain an isoform of NO synthase that appears functionally identical to endothelial NO synthase, including its sensitivity to shear forces [12]. Although the precise mechanism for shear-dependent RBC NO synthase activation remained unclear for some time, this observation was tantalizing given the potential intracellular and extracellular targets for endogenous NO. Preliminary clues exist for both vascular and intracellular targets but conclusive evidence remains lacking. While these shear sensitive processes had thus been known for some time, intriguingly, the molecular identity of the sensor facilitating transduction of mechanical stimuli into biochemical responses was unknown. A breakthrough observation by Cahalan and colleagues [13] provided compelling evidence that Piezo1, a nonselective cation channel, was present in RBC membranes. Their works led to a mechanistic understanding of the well characterized, but incompletely explained, Gárdos effect: while it was understood that Ca^2+^ accumulation in RBC results in K^+^ efflux and dehydration of the cell [14], Cahalan and colleagues [13] delivered vital evidence that Piezo1 operates as the primary transducer of mechanical stimuli into electrochemical signals. Recent works have extended these findings, and demonstrated that mechanical strain of RBC membranes and the resultant Piezo1 activation (Figure 1, inset) appears primal in endogenous NO generation and even cell senescence [15,16], which suggests further unresolved mechanosensitive processes in RBC physiology likely await. Collectively, the contemporary view of RBC physiology has undergone a paradigm shift: rather than being viewed as passive oxygen carriers, complex functional contributions of RBC to organismal homeostasis are on the horizon, particularly those driven by mechanosensitive processes.

## The erythrocyte environment and general considerations

Mechanosensing (and consecutive response) appears a fundamental aspect of RBC physiology. Effective responses to the sensation of mechanical stimuli are transduced by various players, among which ion channels are the most rapid, illustrated, for example, in the generation of action potentials within excitable cells. There is evidence of only seven different ion channels being present in mature human RBC [17]. Three of these channels have been claimed to possess mechanosensitive properties: the NMDA receptor [18–20], the transient receptor potential channel of vanilloid type 2 (TRPV2 [21,22]), and Piezo1 [13,23,24]. While proteomic studies successfully identified Piezo1-peptides in RBC lysates shortly after its discovery [25,26], limited sensitivity delayed successful detection of TRPV2 [27,28] and precluded that of NMDA receptors altogether. All of these proteins are nonselective cation channels that each exhibit unique gating behavior. Whereas NMDA receptors and TRPV2 channels have clearly established agonist-dependent gating (e.g. glutamate for the NMDA receptor, cannabinoids for TRPV2), evidence supporting mechanosensitive gating mechanisms is less convincing [29,30]. For Piezo1, on the other hand, there is a very clear mechanosensing mechanism. Although NMDA receptors and TRPV2 channels might provide redundant mechanosensing, or operate downstream of Piezo1, current evidence suggests that Piezo1 is the primary mechanosensitive channel in RBC, and thus the central topic of this review.

*In vivo*, RBC are suspended in blood plasma, a complex fluid phase containing ~ 60–80 g/L proteins, ~4.5–8.5 g/L lipids, ~8 g/L salts, up to 1 g/L glucose, and dozens of other substances like organic acids. These constituents are essential for the fundamental physiological function of blood, which includes transport and removal of metabolites [31], coagulation [32], communication within the body and its immune response [33] – all of which remain active fields of research. Nonetheless, RBC are mostly investigated *ex vivo*; that is, in many fields the plasma is replaced with a solution of well-defined content and physical properties. Traditionally, maintenance of RBC homeostasis within such new solutions was determined by simply observing changes in cell morphology induced by non-physiological pH or osmolarity. Therefore, the simplest and most widely used solution for suspending RBC is phosphate buffered saline (PBS), containing essential salts in physiological concentrations and a phosphate buffering system to maintain physiological pH. While this suspension keeps RBC alive, it can be likened to banishing cells to solitary confinement without food and a means for communication. Further, cells are typically suspended at very low hematocrit, reducing effect of neighboring cells and bulk fluid viscosity on producing mechanical cues. Obviously, serious experiments, especially when performed over longer time periods, involve suspending RBC in more sophisticated media with numerous additives and complementary substances which better mimic plasma, and occasionally even whole blood. To put it lightly, *in vitro* investigations of RBC typically differ profoundly from the *in vivo* environment, leaving large gaps in our ability to translate experimental evidence into physiological insights. Hence, when it comes to fine-tuned processes such as transduction of mechanical cues through Piezo1 or other pathways and regulation, care needs to be taken when transferring insights generated *in vitro* into the physiological context.

## Mechanosensing mechanisms in erythrocytes

Current suggestions that Piezo1 is the sole sensor of mechanical forces acting as an “on-off switch” of downstream signaling process are most likely an incomplete picture of the true complexity of RBC mechanosensing. Unlike most cells, RBC are constantly subjected to varying shear forces that ultimately contribute to mechanical distortion of their membrane, raising questions about how mechanosensitivity in such a “noisy” environment is regulated. Further understanding of RBC mechanosensitivity fine-tuning, possibly through modulation of Piezo1-properties *via* membrane-interactions or auxiliary signaling processes, will likely progress contemporary perspectives of such regulatory processes and highlight the complexity of RBC physiology.

In both prokaryotes and eukaryotes, two opening models are generally accepted to underlie the gating mechanisms of mechanosensitive channels. The first is referred to as the “force from lipids” (FFL) principle and the second as the “force from filament” (FFF) principle [34]. The FFL model implies that the mechanical force resulting from tension within the membrane lipid bilayer is sufficient for gating of mechanosensitive channels, without the need for other cellular components [35]. Conversely, the FFF paradigm, also called the tether model, suggests that the channel is directly physically linked to either components of the extracellular matrix or submembrane structures such as the cytoskeleton, which are in turn responsible for mechanical gating [36].

In the context of the FFL model, the structure of channels like Piezo1 itself contributes to its mechanosensitivity. Indeed, Piezo1 forms a bowl-shaped trimer within the membrane, comprising a central ion-conducting pore with an extracellular cap and three curved, non-planar, blades with intracellular beams [37]. These beams may explain the extraordinary sensitivity of Piezo1 to lateral membrane tension. It is generally accepted that the half maximal pressure required for activation of Piezo1 is ~30 mmHg [24]. Although it is difficult to convert force or pressure into membrane tension, since it depends on the geometry of the object to which the force is applied, it is generally accepted that 30 mmHg of pressure corresponds to a tension of 4–4.5 pN/m [38]. This aspect is particularly true for the structure of RBC, where the biconcave shape, and the various transitional shapes these cells assume in circulation, place strong constraints on membrane tension, which are rapid but ultimately compatible with the kinetics of activation and inactivation of the Piezo1 channel. Briefly, Piezo1 channels can exist in open, closed, or inactive states [39]. The open active state allows for ion flux, while the closed state renders Piezo1 impermeable, and the inactive state precludes mechanical activation entirely. Entering the inactive state after channel opening is important because it prevents excessive ion flux. Importantly, recent work suggests the existence of several distinct Piezo1 sub-states [40]. The complex mechanisms underpinning transitions between states are incompletely resolved but represent a highly active area of investigation [41,42] with a recent study resolving the structure of Piezo1 in an intermediate open state [43]. Tension applied through micropipette aspiration is generally sufficient to trigger sporadic reversible Ca^2+^-entry [13] which may be sufficient to subsequently activate the Gárdos channel [44]. This is exemplified *in vivo* by intravital recordings of Fluo-4 stained RBC in mice or in microfluidic systems [23].

Within the RBC membrane, Piezo1 is able to diffuse rather freely, as shown directly by immuno-gold particle tracking of Piezo1-molecules in live RBC [45]. Accordingly, at least within the resting RBC geometry, Piezo1-molecules preferentially localize to the “dimple” region [46], implying a preference for areas of high curvature [47]. Alternatively, given that Piezo1-molecules are hypothesized to induce local curvature themselves [37,48,49] through lipid redistribution [50], Piezo1 May contribute to the formation of the characteristic RBC biconcave morphology in the absence of flow. Given the involvement of non-muscle myosin IIa (NMIIa) in maintaining resting RBC biconcavity [51,52], it is tantalizing to speculate that interplay may exist between intracellular forces generated by NMIIa and Piezo1-channels [53] – potentially through dynamic co-localization of Piezo1 with NMIIa at cytoskeletal membrane complexes. A direct interaction between these proteins would provide strong experimental evidence for the FFF model being relevant to RBC, although it has not yet been provided. Convincing direct experimental evidence for Piezo1-interacting partners that support the FFF model has not yet been provided in any cell type but would represent a significant finding. It is noteworthy that the phosphorylation status of NMIIa, which regulates its contractility [54], differs between reticulocytes and mature RBC [55]. NMIIa is a key element in the remodeling/maturation of reticulocytes induced by circulatory shear stress, further pointing to a potential coupling of NMIIa and Piezo1 within RBC. The potential coexistence, at least in RBC, of Piezo1-opening mechanisms following both the FFL and FFF models is further implied by the chemical activation of Piezo1 by the agonist Yoda1 [46], where diffusion of Piezo1 within the membrane is not only dictated by the lipid microdomains surrounding Piezo1 and their rearrangement, but also the dynamics of the spectrin mesh cytoskeleton [56]. Indeed, there is evidence supporting lateral linkage of the RBC cytoskeleton with cholesterol-enriched microdomains in the lipid bilayer, further supporting a possible convergence of the FFL and FFF activation mechanism in RBC [57]. It remains to be seen, however, whether dynamic re-distribution of Piezo1 also occurs during mechanical activation.

The RBC membrane is highly enriched with cholesterol, when compared with other cell types [58]. It is clear that cholesterol plays a key role in membrane fluidity/rigidity and therefore in the magnitude of mechanical forces required to activate RBC-Piezo1. An optimal concentration of cholesterol appears required for the normal functioning of the Piezo1 channel, given that either addition or removal of cholesterol has been shown to modulate Piezo1 mechanosensitivity both *in silico* [59] and *in vitro* [60]. Further, removal of cholesterol has been shown to delay channel inactivation and exacerbate the slow-inactivating phenotype of a Piezo1-mutant underlying hereditary xerocytosis (HX [61]). An in-depth analysis of cholesterol-binding domains within the Piezo1 sequence has identified 19 evolutionary conserved sequences found predominantly on the intracellular domain (CRAC motif) and 40 that tend to embed on the outer membrane (CARC motif). Of these, 8 CRAC and 15 CARC sequences overlap with residues with significant cholesterol interactions in Piezo1 modeled structure [59]. The importance of the lipid environment, or membrane basal tension, in the resting activity of RBC-Piezo1 is further exemplified by the functional gains associated with pathological Piezo1 mutations. Indeed, the first discovered mutations initially indicated that the phenotype of RBC dehydration was linked to an alteration in the intrinsic inactivation mechanisms of the channel [62]. When Piezo1 channels with mutations linked to a pathological hematological phenotype were expressed in a heterologous system (i.e. independent of the unique mechanical environment of RBC) Piezo1 inactivation kinetics appeared normal [63]. This evidence should be considered in parallel with observations suggesting that RBC provide a specific environment for Piezo1 that alters or significantly slows down its rapid inactivation mechanism, thus conferring greater importance to the phenomenon of deactivation, which is a different mechanism from that responsible for inactivation [64]. Such observations therefore imply that many pathologies or conditions affecting the rigidity or stiffness of RBC likely also affect the mechanosensitivity of Piezo1 in the membrane, modulating subsequent signals.

## Cellular modulators of erythrocyte Piezo1-signals

Piezo1 instigates intracellular signaling by facilitating influx of cations in response to mechanical forces exerted on the RBC membrane. The structure of Piezo1-trimers comprises a central ion pore module that spans the lipid bilayer, anchored by three beams, resembling the structure of a propeller. Piezo1 channels directly influence curvature of the membrane, which appears central to their activation mechanism. Flattening of the channel structure is thought to result in removal of the cap from the pore to initiate ion permeation, although this mechanism requires further evaluation [49]. Despite detailed studies of purified mouse Piezo1, either enriched in suspension or incorporated into lipid microvesicles, its structure remains incompletely resolved [49]. Publicly accessible structures of human Piezo1 have only recently been provided and contain some clues to explain differences in channel properties between the murine and human isoforms [65]; for example, it appears that human Piezo1 is flatter at basal membrane tension, suggesting it should not be as readily activated by mechanical stimulation as mouse Piezo1.

The transcriptional regulator MyoD (myoblast determination) family – inhibitor domain-containing protein (MDFIC) was recently demonstrated to directly bind the Piezo1-pore module, significantly prolonging inactivation kinetics by stabilizing the open-state following mechanical stimulation through lipid interactions [66]. While proteomic studies of RBC from healthy individuals [67] and those with HX [68] suggest that MDFIC is not abundant in RBC, it remains a possibility that structurally similar proteins may occupy the MDFIC-Piezo1 binding site, thus potentially altering RBC-Piezo1 inactivation kinetics. Presumably, RBC require rapid inactivation of Piezo1 following capillary transit to prevent excessive accumulation of cytosolic Ca^2+^ [15] – a process that MDFIC-like proteins, if present, would likely influence. Further, *β*-amyloid peptide was recently suggested to directly interact with Piezo1, altering channel properties in patch-clamp experiments [69]. Given that circulating *β*-amyloid appears to be stored in platelets and released in response to various stimuli [70], this novel interaction may be of relevance to RBC-Piezo1, although independent confirmation of this observation, and mechanistic data supporting a potential interaction, have not yet been provided. Similarly, a recent report shows that insulin, which is structurally similar to *β*-amyloid [71], facilitates Piezo1-activity in RBC [72], although likely not through a direct interaction. Collectively, while modulatory Piezo1-interacting proteins have been proposed, so far only MDFIC is supported by solid evidence. The emerging Piezo1-interactome has recently been mapped using a covalent proximity labeling approach [73], albeit this approach was limited to human embryonic kidney cells. Nonetheless, a library of potential Piezo1-interacting proteins was produced, spanning functional domains including cell adhesion, cellular signaling, proteolysis, and antigen-presentation. It will be critical to thoroughly validate whether these are true biochemical interactions with functional consequences for Piezo1 and Piezo1-dependent processes to unlock the therapeutic potential these unique targets may provide.

There is some evidence supporting that the number of functional Piezo1-copies per RBC declines with increasing *in vivo* cell age [15]. The mechanisms for this remain unclear, although it is known that RBC shed membrane vesicles during their lifespan, increasing cell density particularly toward the end of their circulatory lifespan [74]. RBC-derived vesicles, known to be released during mechanical force exposure [75] and in response to increased intracellular Ca^2+^ concentration [76], have been shown to contain Piezo1 [77]. Degradation of Piezo1 due to circulatory stresses or sequestering away from the membrane are possible alternative explanations. We speculate that this loss of Piezo1 May serve to balance the declining metabolic capacity of glycolysis-dependent RBC required for keeping intracellular Ca^2+^ low despite the immense concentration gradient across the cell membrane.

RBC-Piezo1 activation, both by mechanical forces and the chemical agonist Yoda1, has been shown to be sensitive to thiol oxidation induced by the agent diamide [16]. Diamide induces membrane rigidification in RBC through formation of di-sulfide bridges, restricting mobility of membrane proteins and lipids [78,79]. Interestingly, thiol oxidation of purified Piezo1 restricts movement of the cap, which appears necessary for ion transduction, thus temporarily inhibiting Piezo1-mediated currents [80]. Whether cross-linking within Piezo1 sub-domains, increased membrane rigidity, or bridge-formation between Piezo1 and other RBC membrane proteins/lipids underlies the observed restriction in ion flow remains uncertain.

**Figure 2.**
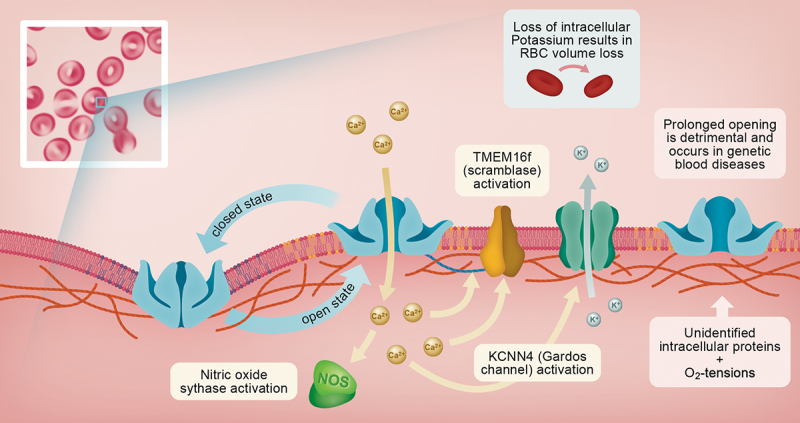
Red blood cells (RBC) provide a unique physical environment for Piezo1-dependent mechanosensing due to exceptional cellular flexibility and morphological geometry with complex curvatures. Membrane lipids and cytoskeletal elements likely contribute to dynamic channel gating during cellular deformation. Potential Piezo1-interacting proteins are indicated as “unidentified intracellular proteins.” Cellular (e.g. intracellular protein organization), genetic (e.g. mutations in the *PIEZO1*-gene causing hereditary blood disorders), mechanical (e.g. heterogenous vessel geometry) and chemical (e.g. oxygen tensions) effectors encountered by RBC during transit of the vasculature modulate Piezo1-gating, although the underlying mechanism are poorly understood.

Downstream targets of Piezo1-mediated calcium-signaling include lipid scramblases. While reversal of lipid asymmetry within the RBC membrane has historically been associated with senescence [81], the molecular identity of the RBC calcium phospholipid scramblase (CaPLSase) was only recently revealed as TMEM16F [82]. Liang and colleagues [82] observed increased coupling of Piezo1 and TMEM16F in human RBC obtained from those with HX. Further, they also provided evidence that phosphatidylserine externalization following Piezo1 activation required TMEM16F, at least in a TMEM16F knock-out murine model. The precise interactions and determinants for coupling between TMEM16F and Piezo1, particularly in healthy humans, however, await further evidence. A summarized integration of the known and predicted intracellular processes involved in Piezo1 signaling within RBC is provided in Figure 2.

Opening of Piezo1 results in influx of primarily calcium cations (Ca^2+^), although Piezo1 is also permeable to sodium-ions and potassium-ions, which impact a growing list of downstream processes. Increased intracellular Ca^2+^-concentration promotes activation of nitric oxide synthases, TMEM16F scramblases, Ca^2+^-dependent potassium channels (i.e. Gárdos channels) serving diverse cellular functions including RBC volume adaptation, RBC adherence and removal from circulation, generation and subsequent export of signaling molecules, and modulation of rheological properties.

Collectively, RBC-Piezo1 function appears sensitive to a range of chemical and physical modifiers, some of which are unique to the RBC environment. For example, the biconcave morphology of enucleated RBC provides a complex membrane curvature, the extensive deformability of which may be exploited for detailed studies of dynamic Piezo1 behavior. The challenges of molecular investigations in RBC, namely the difficulty of genetic manipulation, generating *in vivo* models and inability to culture these cells in meaningful quantities over prolonged periods of time, present a challenge that is actively being addressed, although clinical insights from genetic alterations to RBC-Piezo1 have provided tremendous insights of its relevance *in vivo*.

## Insights into erythrocyte Piezo1-function from genetic variants

Along this line, it is not completely clear in which *in vivo* situations RBC-Piezo1 is activated. The shear forces experienced by circulating RBC in larger blood vessels are probably not strong enough to activate Piezo1, at least in healthy individuals (Figure 1, inset); however, there is evidence supporting that the strong mechanical deformation of the cell membrane during passage of capillaries [23] or the sinusoidal slits in the spleen [15] does activate Piezo1. Experimental tools to assess this *in vivo* are extremely limited, although conclusions can be drawn from genetic variants of Piezo1. Piezo1 is an exceptionally large protein (human isoform: 2521 amino acids/monomer), thus, it presents with increased probability of mutagenesis. Indeed, a large variety of different amino acid substitutions have been reported and linked to adverse clinical outcomes like HX, summarized in detail in [36]. It appears that Piezo1-mutations with clinical significance are strongly clustered around the central ion conducting pore, potentially affecting Piezo1-function by interfering with normal transitions between open, closed, and inactive states. Activated Piezo1, as a nonselective cation channel, allows entry of Ca^2+^ into the cell [13]. Although the opening of Piezo1 *per se* is transient in healthy cells, the high 20,000-fold gradient of Ca^2+^ between the RBC cytosol and surrounding plasma facilitates an influx of cytosolic Ca^2+^ that is sufficient for Gárdos channel (KCNN4, K_Ca_3.1) activation [23]. The consecutive K^+^-loss (associated with Cl^−^-loss, for electroneutrality) results in water efflux and thus mild dehydration [83]. Gain-of-function variants of Piezo1 associated with HX [84] cause HX-RBCs to be dehydrated [62], which has been consistently linked to delayed Piezo1-inactivation in HX-RBC [85], accelerating accumulation of cytosolic Ca^2+^ across the RBC lifespan with every capillary transit. Due to the nonselective nature of the Piezo1-cation gate [86,87], Na^+^ may enter RBC alongside Ca^2+^, and persistent opening could precipitate cell swelling instead of shrinkage, which has been shown in RBC *in vitro* by stimulating TRPV2 with cannabinoids [88]; although evidence of this feasibly occurring *in vivo* has not yet been provided.

RBC are non-excitable cells (in the classic sense) with a resting membrane potential of approximately −10 mV [89], which may have implications for Piezo1 channel behavior *in vivo*, depending on its interplay with other channels and transporters. Piezo1-overexpression induced in N2A and HEK293T cell systems yields rapid inactivation kinetics of ~16 ms when measured using whole-cell patch clamping with a holding potential of −80 mV [24]. Interestingly, inactivation kinetics are significantly prolonged ~ 2-fold when the holding potential is increased to −40 mV, and prolonged by at least one order of magnitude at positive holding potentials [24]. This apparent voltage-sensitivity of Piezo1 is thought to be linked to outward permeation of ions and appears to be regulated by similar residues that are implicated in HX pathophysiology, suggesting its clinical relevance [41,90]. For example, *ex vivo* experiments show that once Ca^2+^-influx is sufficient for activation of Gárdos channels, efflux of K^+^ induces hyperpolarisation, thus exacerbating Ca^2+^-entry *via* Piezo1 [91]. While hyperpolarisation promotes Piezo1-inactivation in most cell types [92], it appears that rapid Piezo1 inactivation is prevented in RBC [64]. Upon removal of the mechanical stimulusit i, Piezo1-dependent influx diminishes. Delayed inactivation kinetics and specific Piezo1-deactivation mechanisms affected by mutations observed in HX may thus be of profound severity for RBC.

Currently, there is discussion about a potential channel interaction amplifying initial Ca^2+^-influx into RBC *via* Piezo1 [93]. The stochastic activity of the low number of Gárdos channels within RBC results in membrane potential flickering under resting conditions, generating so-called “pseudo action potentials” [91]. This could directly activate the voltage-activated Ca^2+^ channel Ca_V_2.1 [94], but also acutely modulate Piezo1-inactivation as discussed above. In any case, following Piezo1-dependent Ca^2+^-entry and K^+^ loss, the original ion equilibrium is restored. This is reached by ATPase activity of both the Ca^2+^-pump (PMCA [95]) and the Na^+^/K^+^-pump (ATP1A1 [96]), requiring ATP, and hence glucose. Given RBC do not contain mitochondria, they are exclusively dependent upon anaerobic glycolysis for ATP-production [97]. This requirement of ATP to fuel ion pumps also explains the increased glucose consumption rate observed in HX-RBCs [98], and the accelerated metabolism of stretched RBC [99]. In case Ca^2+^ extrusion does not compensate for Ca^2+^ entry, RBC may undergo a suicidal death [15]. This happens at the end of the RBC lifetime or when RBC integrate with blood clots [100], but may happen at increased rates in HX and/or sickle cell, contributing to decreased circulating RBC counts, and thus the development of anemic conditions [101].

Collectively, tremendous insight into Piezo1-function within RBC has been gained from studying cells from donors with a range of Piezo1-variants, enabling a glimpse into the *in vivo* significance of normal Piezo1 function. Significant relevance has also been ascribed to RBC-Piezo1 in the special case of sickle cell disease (SCD), and recent computational modeling studies have enabled tracking of Piezo1-dependent processes across the RBC lifespan.

## Hyperactive deoxy-Piezo1 – The root cause of sickle cell disease complications?

SCD is caused by the homozygous inheritance of the abnormal hemoglobin, HbS. Upon deoxygenation in the venous circulation, HbS polymerizes, deforming RBC into sickle-like shapes. Sickle cell deformation, in turn, permeabilizes the cells to Ca^2+^ *via* Piezo1 channels (i.e. the historical P_sickle_ permeability pathway [102]), triggering a Ca^2+^-dependent dehydration cascade driven by the outward electrochemical K^+^-gradient. This mechanism operates with particular intensity in a subpopulation of sickle RBC, the irreversibly sickled cells (ISC). This sickle cell subtype is considered responsible for vaso-occlusion, the root cause of organ failure and pain crisis in SCD [103,104].

A well-accredited model of RBC homeostasis, tightly constrained by a large body of validated experimental observations on sickle cells, was recently applied to investigate the experimentally inaccessible ISC life cycle *in vivo*, and the role of Piezo1 channels in ISC formation [105]. Unlike in normal RBC, Piezo1 channels of sickle cells entering venules remain open for the duration of each deoxy transit, the result of a block of the normal spontaneous inactivation process, a block fully reversible when sickle cells reenter the oxygenated arterial streams [102,106,107]. The inactivation block is a common response for all three main sickle cell subtypes (discocytes, fetal hemoglobin carrying sickle cells termed F-cells and ISCs), an effect attributed to interactions between Piezo1 and the deoxy-hemoglobin fibers responsible for the familiar sickle-like RBC deformations [108–110]. Sickling progressively dehydrates all sickle cells and reduces their lifespans, to ~15 days for discocytes, ~45 days for F-cells [111], but maximally to 4–7 days for ISCs [112,113], differences the model reproduces by varying the mean overall cell conductance attributed to deoxy-Piezo1 channels in the venous circulation [114]. For ISCs to rapidly dehydrate to a hyperdense pathogenic state within a day or so in the circulation, as documented [112], the cell conductance mediated by deoxy-Piezo1 channels had to be set at least tenfold higher than that in the other sickle cell subtypes [105]. This result showed that the increased conductance mediated by deoxy-Piezo1 channels in ISCs is the root cause of ISC formation and sickle cell disease. Unraveling the mechanism of deoxy-Piezo1 hyperactivity in ISCs has become central to the search for alternative, Piezo1-related therapies in SCD [115,116].

## Concluding remarks and outlook

Collectively, astounding progress has been achieved in resolving open questions in the field of RBC biology following the discovery of Piezo1. Areas of intense focus have included investigations of how Piezo1 impacts intracellular signaling processes and the RBC lifespan, characterizing how various mutations in the *PIEZO1-*gene affect RBC properties and precipitate clinically relevant phenotypes (e.g. anemia), and Piezo1-function in sickle cells. It is important to emphasize that most of these studies are conducted under *ex vivo* experimental conditions which are necessarily limited and may not fully reflect the complex *in vivo* environment. Thus, insights gained from these experiments should be extrapolated with caution and may require reconciliation when advanced experimental techniques become available in the future.

It is also clear that we are only scratching the surface of Piezo1-dependent signaling pathways and mechanisms within RBC. As outlined in this work, there are numerous gaps and currently experimentally inaccessible questions. For example, the complexity of introducing conditional genetic alterations to enucleated RBC, although recently successful [117,118], hinder the development of *in vivo* models. Further, the unique cellular environment of RBC limits translatability of observations from heterologous cell systems, requiring independent validation using RBC. Albeit there is significant progress in investigating RBC with automated patch clamp systems [119,120], high-fidelity micropipette aspiration techniques [15,121] and novel microfluidic platforms [122–124], difficulty of generating reliable and representative electrophysiological recordings from RBC ion channels remains. These challenges notwithstanding, it has become clear that RBC are not biologically inert, rather, these cells contain surprisingly complex molecular signaling networks which appear fundamentally integrated with mechanical cues, including but not limited to that downstream of Piezo1.

## Data Availability

Data sharing is not applicable to this article as no new data were created or analyzed in this study.
